# A Combined Experimental and Computational (DFT, RDF, MC and MD) Investigation of Epoxy Resin as a Potential Corrosion Inhibitor for Mild Steel in a 0.5 M H_2_SO_4_ Environment

**DOI:** 10.3390/polym15081967

**Published:** 2023-04-21

**Authors:** Rachid Hsissou, Khadija Dahmani, Anouar El Magri, Abdelfettah Hmada, Zaki Safi, Nadia Dkhireche, Mouhsine Galai, Nuha Wazzan, Avni Berisha

**Affiliations:** 1 Laboratory of Organic Chemistry, Bioorganic and Environment, Chemistry Department, Faculty of Sciences, Chouaib Doukkali University, BP 20, El Jadida 24000, Morocco; 2 Laboratory of Organic Chemistry, Catalysis and Environment, Department of Chemistry, Faculty of Sciences, Ibn Tofail University, BP 242, Kenitra 14000, Morocco; khadija.dahmani@uit.ac.ma; 3 Euromed Polytechnic School, Euromed Research Center, Euromed University of Fes, Fès 30000, Morocco; a.elmagri@ueuromed.org; 4 Laboratory of Advanced Materials and Process Engineering, Faculty of Sciences, Ibn Tofail University, BP 242, Kenitra 14000, Morocco; abdelfettah.hmada@uit.ac.ma (A.H.); nadia.dkhireche@uit.ac.ma (N.D.); galaimouhsine@gmail.com (M.G.); 5 Chemistry Department, Faculty of Science, Al Azhar University-Gaza, Gaza BP 1277, Palestine; zaki.safi@gmail.com; 6 Chemistry Department, Faculty of Science, King Abdulaziz University, BP 42805, Jeddah 21589, Saudi Arabia; nwazzan@kau.edu.sa; 7 Department of Chemistry, Faculty of Natural and Mathematics Science, University of Prishtina, 10000 Prishtina, Kosovo; avni.berisha@uni-pr.edu

**Keywords:** TGP epoxy resin, MS/0.5 M H_2_SO_4_, corrosion inhibitor, PDP/EIS techniques, SEM/EDS analyses, DFT calculation, MC and MD simulations

## Abstract

In this work, a tetrafunctional epoxy resin entitled 2,3,4,5-tetraglycidyloxy pentanal (TGP) was tested and investigated as a potential corrosion inhibitor for mild steel (MS) in 0.5 M H_2_SO_4_ solution. The corrosion inhibition process for mild steel was employed alongside various techniques, such as potentiodynamic polarization (PDP), electrochemical impedance spectroscopy (EIS), temperature effect (TE), scanning electron microscopy (SEM), energy-dispersive X-ray spectroscopy (EDS) and theoretical approaches (DFT, MC, RDF and MD). Further, the corrosion efficacies obtained at the optimum concentration (10^−3^ M of the TGP) were 85.5% (EIS) and 88.6% (PDP), respectively. The PDP results indicated that the TGP tetrafunctional epoxy resin acted the same as an anodic inhibitor type in 0.5 M H_2_SO_4_ solution. SEM and EDS analyses found that the protective layer formed on the MS electrode surface in the presence of TGP could prevent the attack of the sulfur ions. The DFT calculation provided more information regarding the reactivity, geometric properties and the active centers of the corrosion inhibitory efficiency of the tested epoxy resin. RDF, MC and MD simulations showed that the investigated inhibitory resin have a maximum inhibition efficiency in 0.5 M H_2_SO_4_ solution.

## 1. Introduction

Metallic steel is widely employed and investigated in various industrial fields of application, including aeronautics, automotives, railways, metal construction, etc., due to its advantageous availability and excellent mechanical resistance [1,2]. In addition, several methods have been investigated and developed in order to control and prevent the corrosion process in destructive environments, including sacrificial anode protection, galvanization, and the application of polymer composite coatings and/or the use of anticorrosion protection [3,4,5]. One of the most cost-effective and practical approaches is the implementation of organic inhibitory resins. The incorporation of polyepoxide inhibitory resins that possess heterocyclic compounds, epoxy systems, heteroatoms (phosphorus, sulfur, nitrogen and oxygen) and electronegative polar groups is the method used most in order to slow and stop the corrosion of metallic samples in aggressive environments [6,7]. Indeed, they act through an adsorption mechanism on the metal substrate area by forming a protective layer barrier against corrosion in aggressive environments. Among these various compounds, epoxide resins, which result from the reaction between glycidol and halogenated compounds and/or the condensation of epichlorohydrin with compounds, possess mobile protons due to the presence of heterocyclic compounds, aromatic rings and epoxy systems in their structure [8]. Several epoxide resins have been reported and investigated for their anticorrosive protection of metallic samples in aggressive environments [9]. Furthermore, several publications on the evaluation and performance of epoxide resins and their corrosion inhibition activity have investigated the fact that these macromolecules are mostly employed owing to their important inhibitory efficiency [10].

Recently, and based on the great advances made in supercomputers, quantum chemical methods, such as density functional theory (DFT), molecular dynamic simulation and Monte Carlo methods, have become very useful tools in the determination of the molecular structure, electronic structure and molecular reactivity of chemical compounds. Therefore it has become common practice to perform quantum chemistry calculations in order to predict the molecular corrosion inhibition performance and efficiency of different molecules and to understand their inhibitory mechanisms.

In this potential study, an inhibitory resin entitled 2,3,4,5-tetraglycidyloxy pentanal was employed; it performed excellently in terms of providing anticorrosion protection to steel substrate electrodes in 0.5 M H_2_SO_4_, including when analyzed using different characterization techniques, such as potentiodynamic polarization, electrochemical impedance spectroscopy, temperature effect, scanning electron microscopy and X-ray energy-dispersive spectroscopy [11]. To know and understand the interaction mechanism between the investigated inhibitory resin and the surface of the metallic substrates, several global chemical quantum descriptors, such as the electronegativity, the absolute hardness, the electrophilicity index, the softness, the fraction of electrons transferred, the condensed nucleophiles and electrophiles, the Fukui functions, the local softness, the local electrophilicity, the dual Fukui functions, the dual softness and the dual philicity, were used and discussed further. In addition, the DFT method is an important and effective approach to describing the structural and geometric behaviors of a huge number of inhibitors and their role in the protection of several metals [12]. Additionally, the RDF, MC and MD approaches were performed and investigated in order to evaluate the adsorption energies and the adsorption configuration of the inhibitory resin on the Fe(110) area in the simulated aggressive environment [13].

## 2. Experimental Part

### 2.1. Inhibitor Used Material and Characterization Techniques

The inhibitory resin, namely 2,3,4,5-tetraglycidyloxy pentanal, was synthetized, identified and confirmed by Hsissou et al., according to the procedures illustrated several of the previously published studies in this area [6,14]. In addition, the chemical composition of the mild steel (MS) samples used in the experimental section was C (3.66 ± 0.03%), O (22.62 ± 0.06%), S (5.89 ± 0.03%), Cl (0.24 ± 0.01%) and Fe (67.60 ± 0.14%). Moreover, the MS samples with a 1 cm × 1 cm surface were abraded with various grades of sandpaper from 180 to 2000, rinsed with bi-distilled water, washed using acetone, and then dried at room temperature. Then, to protect the MS surface, a TGP concentration range of 10^−3^ to 10^−6^ M in 0.5 M H_2_SO_4_ solution was maintained in order to obtain the potentiodynamic polarization curves (PDP) and electrochemical impedance spectroscopy (EIS) measurements. In addition, the electrochemical measurements were investigated using a Potentiostat/Galvanostat/ZRA (Gamry apparatus). Furthermore, the electrochemical cell was composed of the mild steel, which was used as the work electrode, the platinum, which was used as the counter electrode, and the saturated calomel electrode, which was used as the reference electrode. The open circuit potential (OCP) was maintained for 30 min in each assay after reaching the equilibrium state. Then, the PDP plots were measured at 0.3 mV/s when the potential range was between −900 and −100 mV. The EIS measurements were carried out when the OCP was in the frequency range of 100 KHz to 0.1 Hz with an AC voltage of 10 mV. In addition, the surfaces of the MS samples immersed in 0.5 M H_2_SO_4_ solution were uninhibited and inhibited with 10^−3^ M of TGP; this was performed using a scanning electron microscope and energy-dispersive X-ray spectroscopy. In addition, the SEM technique was investigated for its potential ability to create images of the uninhibited and inhibited mild steel surface in the presence of TGP at 10^−3^ M, using a JEOL-JSM-5500-type microscope. Moreover, EDS analysis was used in order to obtain the elemental chemical composition. Furthermore, the measurement conditions were as follows: the magnification was x2000, the process time was T4, the acceleration voltage was 20.00 kV, the measurement was first, the real time was 38.74 s, the live time was 30.00 s, the count rate was 29,643.00 CPS and the dead time was 22.00%. Additionally, the corrosion efficiencies for the potentiodynamic polarization curves and for electrochemical impedance spectroscopy were measured according to Equations (1) and (2), as follows:

(1) $$\%\eta_{\mathrm{PDP}}=\frac{i_{\mathrm{corr}}^{0}\;-\;i_{\mathrm{corr}}}{i_{\mathrm{corr}}^{0}}\times 100$$

(2) $$\%\eta_{\mathrm{EIS}}=\frac{R_{\mathrm{ct}}\;-\;R_{\mathrm{ct}}^{0}}{R_{\mathrm{ct}}}\times 100\;$$

where $R_{\mathrm{ct}}^{0}$ and $R_{\mathrm{ct}}$ are the charge transfer resistance for the uninhibited and inhibited MS with different concentrations of TGP, respectively. In addition, $i_{\mathrm{corr}}^{0}$ and $i_{\mathrm{corr}}$ are the corrosion current densities for the uninhibited and inhibited MS in the presence of TGP at different concentrations, respectively.

### 2.2. DFT Details

**Scheme 1:**
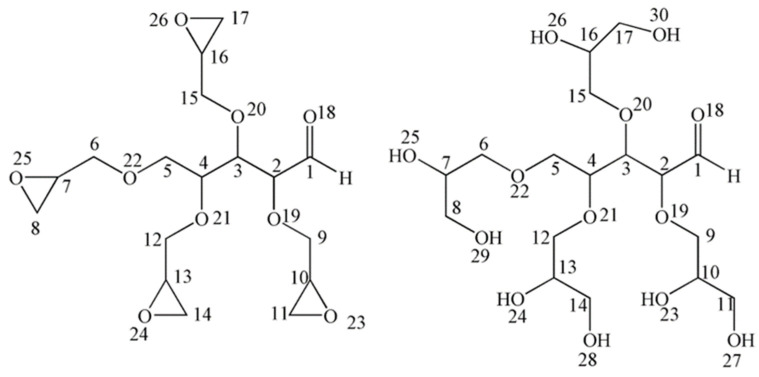
Chemical structures of the TGP (neutral (**left**) and protonated (**right**)). The numbers do not follow the IUPAC system.

In this work, density functional theory (DFT) was used in order to analyze the electronic behaviors of the investigated tetrafunctionnal epoxy resin (TGP) and its protonated form (Scheme 1). Optimization of the proposed geometries of all species was performed by applying Becke’s three-parameter Lee–Yang–Par correlation functional (the B3LYP hybrid functional) [15] alongside the 6-31+G(d,p) basis set. Since the electrochemical corrosion takes place in the aqueous phase and because this study aimed to model the solvent effect, all the calculations were performed in an aqueous environment using the polarizable continuum model (PCM) of solvation [16]. The PCM is a method commonly used to model solvation effects in computational chemistry. It is necessary to consider each solvent molecule as a separate molecule. Two types of PCM have been popularly used: the dielectric PCM (D-PCM), in which the continuum is polarizable, and the conductor-like PCM (C-PCM), in which the continuum is like a conductor. Because corrosion occurs in an aqueous environment, the calculation is more interesting and correlated with the experimental results when the solvent effects are considered. 

The minimum value of the potential energy areas of these samples was found using the absence of imaginary frequencies. The molecular modeling studies were carried out by using Gaussian 09 [17]. Gaussview [18] programs were used to build up the input files, to visualize the highest occupied and lowest occupied molecular orbitals (HOMO and LUMO), and to visualize the electrostatic potential maps (ESP). The energies of HOMO (I = −*E*_HOMO_) and the LUMO (A = −*E*_LUMO_) were investigated in order to illustrate the energy gap (*∆E*_gap_) of the neutral and protonated species (*∆E*_gap_ = *E*_LUMO_ − *E*_HOMO_). Several quantum global reactivity descriptors of the investigated inhibitor, including electronegativity (χ), absolute hardness (*η*), the electrophilicity index (ω), softness (S) and the fraction of electrons transferred (*∆N*), were evaluated in terms of the vertical ionization potential (*Iv*) and vertical electron affinity (*Av*); these were calculated as functions of Equation (3), as follows [19,20,21]:

(3) $$I_{v}\approx E\left(N-1\right)-E\left(N\right)\;\mathrm{and}\;A_{v}\approx E\left(N\right)-E\left(N+1\right)\;\mu_{o}=-\chi =\frac{I_{v}+A_{v}}{2},\;\;\eta =\frac{I_{v}-A_{v}}{2}\;,S=\frac{1}{\eta },\;\omega \;=\frac{\mu_{o}^{2}}{2\eta }\;,\;\Delta N=\frac{\Phi_{Fe}-\chi_{inh}}{2\left(\eta_{Fe}+\eta_{inh}\right)}\;\Delta E_{b-d}=-\frac{\eta_{inh}}{4}$$

In the above set of equations, *E*(*N*), *E*(*N* − 1) and *E*(*N* + 1), and *Φ_Fe_*, $\eta_{Fe}$ and *ΔE*_b−d_, are the total energy of the optimized structures, the cationic and anionic energies at the optimized neutral geometries, the work function (4.82 eV), the hardness of Fe(110) (0.0 eV) and the electronic back-donation energy, respectively [22]. If *ΔE*_b−d_ < 0, the mechanism is energetically favored, otherwise the process is energetically unfavored [23].

Fukui functions can give a useful indication of the local reactivity centers regarding nucleophilic and electrophilic attacks when using the investigated inhibitory resin [24]. To compute the Fukui indices, single-point energy calculations for the neutral, cation and anion radicals that comprise the geometrical structure of the neutral species were performed in order to generate the required wave functions. The condensed nucleophilic ($f_{k}^{+}$) and electrophilic $(f_{k}^{-}$) Fukui functions, the local softness $\left(s_{k}^{\pm }\right)$, the local electrophilicity ($\omega_{k}^{\pm }$), the dual Fukui functions $\Delta f\left(r\right)$, the dual softness and dual philicity, which can give a numerical value for the chemical reactivity of all sites in the molecule, were evaluated using the following expressions (Equation (4)) [25]:

(4) $$f_{k}^{+}=q_{k}\left(N+1\right)-q_{k}\left(N\right)\;f_{k}^{-}=q_{k}\left(N\right)-q_{k}\left(N-1\right)\;s_{k}^{\pm }=Sf_{k}^{\pm }\left(r\right),\;\omega_{k}^{\pm }=\omega f_{k}^{\pm }\left(r\right)\;f_{k}^{2}=\Delta f\left(r\right)=f_{k}^{+}-f_{k}^{-}\;\Delta s_{k}=s_{k}^{+}-s_{k}^{-}\;\Delta \omega_{k}=\omega_{k}^{+}-\omega_{k}^{-}$$

### 2.3. Monte Carlo and Molecular Dynamics Computational

Using the Material Studio package’s Forcite module, the ability of neutral and protonated inhibitory resins to adhere to the area of iron was studied via MC and MD simulations. This was performed in a simulation box (2.4823 nm × 2.4823 nm × 1.824 nm + 3.5 nm vacuum layer) with regular conditions at the edges. For the system being studied, the Fe(110) area with a slab of 15 was chosen; this is because it is the most stable of the three common iron areas [26]. Details about the MS simulations were described previously [27,28]. The slab model in the simulation contained the following: 1 inhibitory resin (in neutral or protonated state), 900 water molecules, and 10 hydroniums plus 5 sulfate ions. In short, these simulations used the COMPASSIII force field [29], according to the following parameters: the NVT ensemble, 295 K, a time step of 1 fs, and a simulation time of 0.8 ns [30].

## 3. Results and Discussion Part

### 3.1. Electrochemical Investigation

**Figure 1:**
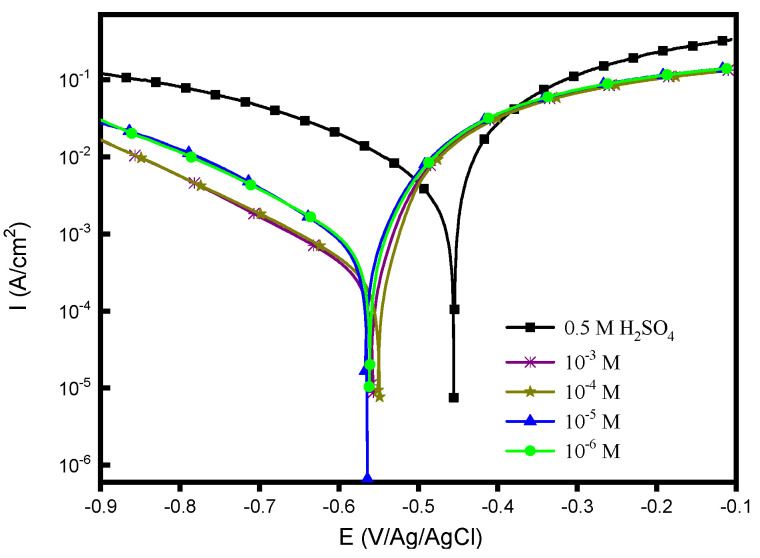
PDP plots for MS in an 0.5 M H_2_SO_4_ environment, uninhibited and inhibited with inhibitory resin at different concentrations.

In order to determine the interaction between the tetrafunctionnal inhibitory resin and the surface of the MS samples uninhibited and inhibited with TGP at different concentrations in 0.5 M H_2_SO_4_ solution at 298 K, PD was used and investigated, as illustrated in Figure 1 and Table 1. The data listed in Table 1 demonstrate that the *i*_corr_ decreases to lower values that ranging between 1850 and 211 µA cm^−2^ with as the TGP concentration increases (from 10^−6^ to 10^−3^ M) [6]. In addition, this diminishes due to the development of a covalent bond between the methoxy group, the ketone function, the oxiran systems and the free electrons of oxygen heteroatoms that possess the vacant iron orbitals of the MS surface [7]. These data demonstrate that the TGP inhibitor, at a low concentration, was able to form a protective layer on the surface of the metallic samples by diminishing the number of active sites. These data also demonstrate that it can be employed as an excellent inhibitory resin for the mild steel electrode in 0.5 M H_2_SO_4_ solution. Additionally, all the corrosion potential displacements (ΔE_corr_) for MS uninhibited and inhibited with different concentrations of TGP (97 to 112 mV) are higher than ±85 mV, indicating that the TGP molecule acted as an anodic inhibitor type [31]. In addition, after carefully examining the results illustrated in Table 1, it is evident that the cathodic and anodic slopes are modified after the addition of the TGP to the aggressive environment, compared to the blank [32]. Further, from the data shown in Table 1, it can be seen that, as the concentration of TGP increases, the inhibitory efficacy increases and reaches a high value of 88.6% at a high concentration, resulting in the TGP potentially being adsorbed on the MS electrode surface.

**Figure 2:**
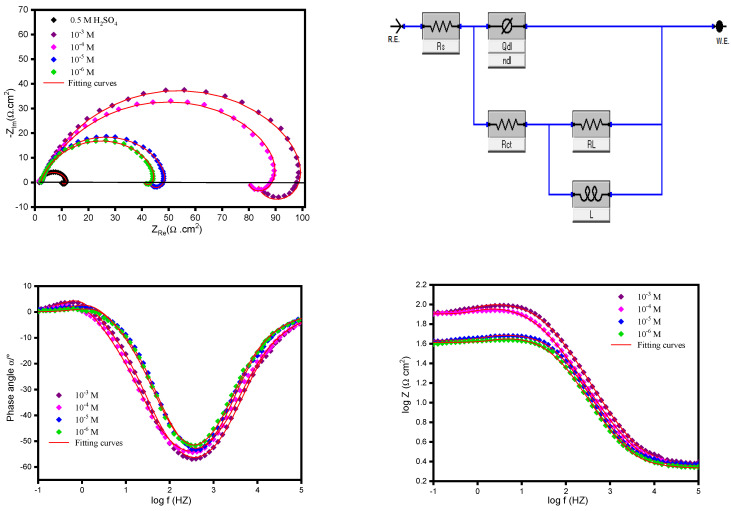
Nyquist and Bode plots and EC for MS immersed in 0.5 M H_2_SO_4_, uninhibited and inhibited by inhibitory resin at different concentrations.

In order to determine and understand the corrosion inhibition of the metallic surface, TGP was employed and investigated. Figure 2 illustrates the Nyquist plots for the anticorrosive protective efficiency of TGP in the 0.5 M H_2_SO_4_ environment, uninhibited and inhibited with TGP at different concentrations. Further, a careful examination of the Nyquist plots reveals that the corrosion processes are the same for both the uninhibited and inhibited MS surfaces. According to the data displayed in Figure 2, the charge transfer resistance and the diameter of the semi-circles increase after the addition of different concentrations of TGP in 0.5 M H_2_SO_4_ solution, which indicates that the TGP can be adsorbed into the metallic substrates by forming a protective layer and by preventing the dissolution of the metal [33]. According to the data illustrated in Figure 2, the Bode plots increase as the concentration of TGP increases, and also decreases according to the frequency. This result implies that the TGP contributes to the formation of a protective layer on the MS surface. In addition, electrochemical data were simulated by using the equivalent circuit (EC), including the electrolyte resistance (*R*_e_), the constant-phase element (CPE(*C*_dl_ and *n*_dl_)), the charge transfer resistance (*R*_ct_) and the inductance element (*R*_L_ and L), as illustrated in Figure 2. A careful examination of the data grouped in Table 2 reveals that the charge transfer resistance values increase as the TGP concentration increases from 11.7 to 81.37 Ω cm^2^, and also that the inductive element values increase from 2.17 to 22.35 Ω cm^2^ for the MS surface uninhibited and inhibited with 10^−3^ M of TGP; this reflects the highest blockage of sulfur ions by using TGP at the MS/0.5 M H_2_SO_4_ interface [34]. These data suggest that the inhibitory resin is able to protect mild steel substrates to an excellent standard by preventing the ion’s diffusion. From the data listed in Table 2, it is evident that a strong agreement exists between the *Q* values and the anticorrosive inhibitory efficacy. For the mild steel electrode, the *Q* values decrease as the TGP concentration increases, but only when compared to the blank; this is due to an increase in the thickness of the protective layer and/or a decrease in the dielectric constant. This protective property is predominantly shown in the differences between the surface states of the metallic substrates in terms of the structure and roughness of the samples after anticorrosive protection. For the inhibitory resin investigated, a more pronounced effectiveness is achieved with 10^−3^ M of TGP, which is in excellent correlation with the data obtained using the potentiodynamic polarization technique. For all concentrations, the *n*_dl_ values for the MS surfaces uninhibited and inhibited with inhibitory resin in 0.5 M H_2_SO_4_ solution are less than 1, resulting in the pseudo-capacitive system of the CPE. These data suggest that the inhibitory resin offers excellent anticorrosion protection compared to other epoxy resins that have been investigated for their potential anticorrosion protection [35].

### 3.2. SEM/EDS Investigation

**Figure 3:**
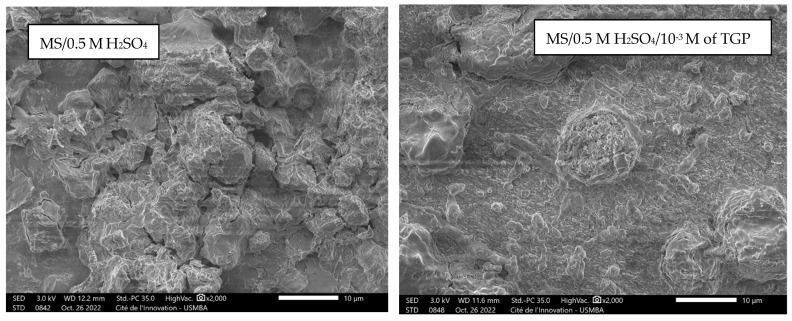
SEM images for uninhibited and inhibited MS substrates immersed in 0.5 M H_2_SO_4_ with 10^−3^ M of inhibitory resin.

**Figure 4:**
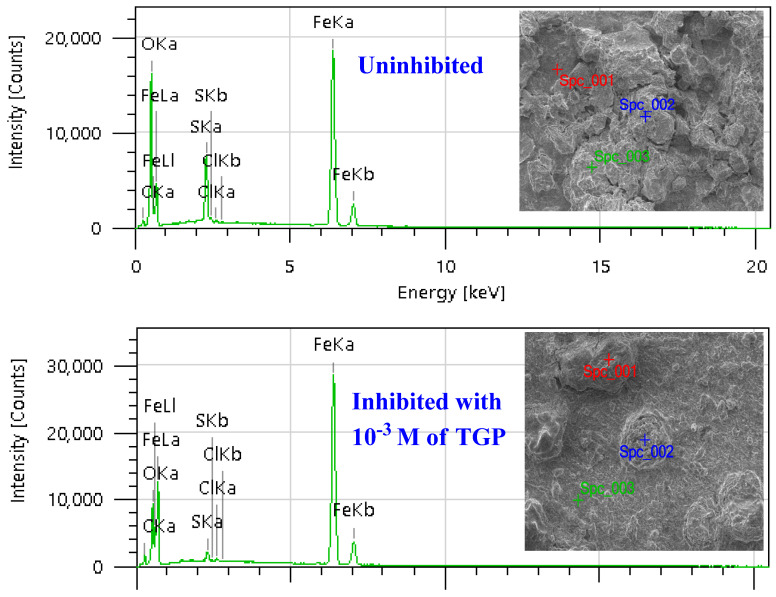
EDS analysis for the uninhibited and inhibited MS electrode area after immersion in 0.5 M H_2_SO_4_ with 10^−3^ M of inhibitory resin.

In order to display and evaluate the adsorption phenomena between the surface of metallic substrates and the TGP employed, SEM and EDS characterization analyses were used and discussed further. Figure 3 displays the SEM morphology of the uninhibited and inhibited MS surfaces immersed in 0.5 M H_2_SO_4_, with inhibitory resin at a higher concentration (10^−3^ M). In addition, the surface of the MS electrode shows cracks, holes and a severe surface. However, the MS samples display the protective layer, and are smooth with fewer cracks and holes with 10^−3^ M of TGP, suggesting that the TGP is a good inhibitor and has a more significant protective effect [6]. Additionally, the EDS characterization data of the uninhibited and inhibited surfaces of the MS samples with 10^−3^ M of TGP in 0.5 M H_2_SO_4_ are shown in Figure 4 and Table 3. Considering the data illustrated in Figure 4 and after a careful analysis of Table 3, it can be remarked that the percentage of Fe present on the MS electrode surface increases from 67.60 to 84.24%, and that the elemental peak of Sulfur (S) decreases from 5.89 to 0.97% after the addition of the inhibitory resin TGP. In addition, the elemental peaks in the content of oxygen and carbon increase with the addition of the 10^−3^ M of TGP (from 9.51 to 34.66% (for oxygen) and from 5.04 to 6.89% (for carbon)) due to the heteroatoms of oxygen and carbon that are present in the inhibitory resin studied; this suggests that the TGP reduces the density of the active sites on the surface of the metallic substrates by forming a protective layer, thus delaying the corrosion inhibition.

### 3.3. Adsorption Study

In order to determine and evaluate the adsorption mechanism mode that functions between the inhibitory resin and the MS surface in the 0.5 M H_2_SO_4_ solution, the experimental results obtained using the PDP technique were evaluated by applying the various adsorption isotherms equations and ascertaining the best fit, which in this case, was the Langmuir adsorption mechanism mode (Figure S1). According to the results found using the PDP plots, it appeared that the TGP was adsorbed on the surface of the MS samples via the formation of a protective layer, which protects the metallic substrates area in an aggressive environment. In addition, Table 4 illustrates the various parameters determined using the PDP technique applied for the studied inhibitory resin. According to data obtained using the PDP technique, the coefficient value (R^2^) is 1, which is in good agreement with the Langmuir adsorption mechanism. In addition, this information indicates that the inhibitory resin TGP could be adsorbed on the MS surface, due to the fact that the best mode could be used in order to develop a certain experience. Moreover, the Gibbs energy variation (Δ*G*_ads_) values for adsorption offer more data concerning the adsorption mechanism performed by the inhibitory resin and the MS surface. Additionally, a Δ*G*_ads_ value that is higher than −40 KJ/mol indicates chemical adsorption. However, a Δ*G*_ads_ value that is less than −20 kJ/mol suggests the physical adsorption mode [6]. Furthermore, the Langmuir adsorption isotherm mode and the Gibbs energy variation for adsorption can be determined by using Equations (5) and (6), as follows [13]:

(5) $$\frac{C_{\mathrm{inh}}}{\theta }=\frac{1}{K_{\mathrm{ads}}}{+C}_{\mathrm{inh}}$$

(6) $$\Delta G_{\mathrm{ads}}=-R\times T\times \mathrm{Ln}\;(55.5\times K_{\mathrm{ads}})$$

where K_ads_, θ, C_inh_, T and R are the adsorption equilibrium constant, the surface coverage degree, the inhibitory resin concentration, the employed temperature and the perfect gas constant, respectively.

A careful analysis of Table 4 reveals that the Δ*G*_ads_ value is equal to −44.74 KJ mol^−1^, which suggests that the TGP inhibitory resin could be adsorbed on the MS area through the chemical adsorption isotherm model. Therefore, the chemical adsorption of the inhibitory resin studied could be due to the nature the bond between the Fe ions and the epoxy systems, and the methoxy function and the free electron of the oxygen heteroatoms present in the TGP inhibitory resin employed.

### 3.4. Temperature Effect and Kinetics Parameters

**Figure 5:**
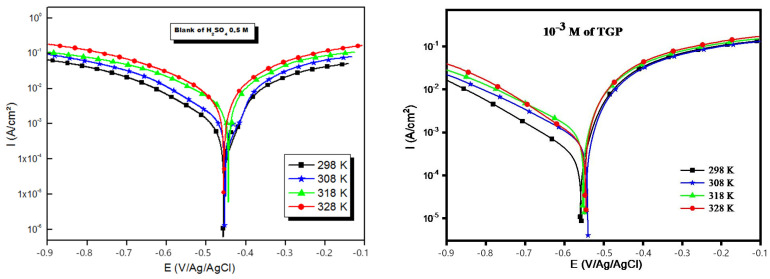
PDP plots of MS uninhibited and inhibited in 0.5 M H_2_SO_4_with inhibitory TGP resin (10^−3^ M) at different temperatures.

In order to determine and evaluate the adsorption mechanism, the temperature effect of the system was one of the essential factors used to study the corrosion inhibition of an uninhibited and inhibited steel electrode in a 0.5 M H_2_SO_4_ medium with an optimal concentration of TGP at different temperatures. In addition, in order to study the temperature effect on the inhibitory resin employed, potentiodynamic polarization analysis was carried out at varying temperatures, as follows: 298, 308, 318 and 328 K. Figure 5 illustrates the temperature effect of the PDP plots obtained in an aggressive environment for MS uninhibited and inhibited with 10^−3^ M of inhibitory resin and tested at different temperatures. The different electrochemical parameters obtained using the PDP curves for different temperatures are displayed in Table 5. A careful examination of the data illustrated in Table 5 reveals the difference in the corrosion potential between the MS uninhibited and inhibited with 10^−3^ M at a TGP concentration higher than 85 mV/SCE, for all the temperatures investigated. Further, it is evident that the cathodic slops within 10^−3^ M of TGP at different temperatures were higher compared to those of the acidic environment only; these data indicate that the TGP studied acted as an excellent anodic-type inhibitor. According to the data listed in Table 5, it is evident an increase in the temperature led to an increase in the corrosion current densities and to a decrease in the inhibitor efficiencies. This result confirms that the mild steel electrode can be dissolved by increasing the temperature. Additionally, these decreases could be explained by the phenomenon that sees the adsorption of the TGP on the surface of the metallic substrate.

In order to determine the activation energies of the corrosion process of the metallic electrode surface when it is plunged in an aggressive environment (0.5 M H_2_SO_4_) at 10^−3^ M of employed inhibitory resin, the Arrhenius formula between the *i*_corr_ values and the temperature applied was used and investigated according to Equation (7). Additionally, an alternative formula for the Arrhenius relationship was investigated in order to calculate the activation standard enthalpy (∆*H*_a_) and the activation standard entropy (∆*S*_a_), according to Equation (8), as follows [36]:

(7) $$i_{\mathrm{corr}}=\mathrm{Aexp}\left(-\frac{E_{a}}{RT}\right)$$

(8) $$i_{\mathrm{corr}}=\frac{RT}{hN}\exp\left(\frac{\Delta S_{a}}{T}\right)\exp\left(-\frac{\Delta H_{a}}{RT}\right)$$

where A, *h*, *N*, and *R* denote the Arrhenius constant (pre-exponential factor), the Planck number, the Avogadro number and the perfect gas constant, respectively.

**Figure 6:**
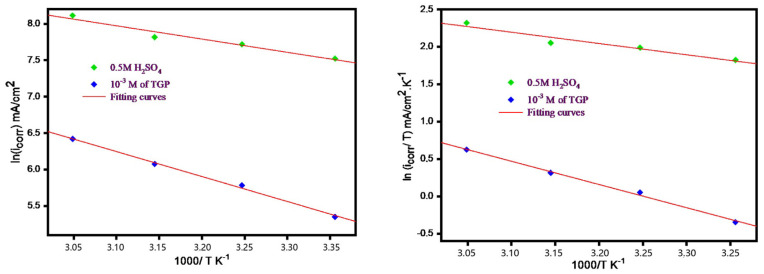
Ln(*i*_corr_) as a function of 1000/T (**left**) and Ln(*i*_corr_/*T*) as a function of 1000/T (**right**) curves for MS uninhibited and inhibited in 0.5 M H_2_SO_4_ with 10^−3^ M of TGP.

Furthermore, the activation energy values were measured using the slopes of the lines. In addition, Figure 6 illustrates the graphical representations of ln(*i*_corr_) as a function of 1000/T for MS uninhibited and inhibited with the investigated inhibitory resin at different concentrations. Table 5 illustrates the activation energy values calculated using the straight lines of the Arrhenius equation for MS uninhibited and inhibited with TGP. According to the results reported in several previously published studies, when the activation energy of the studied inhibitory resin is greater than that of the 0.5 M H_2_SO_4_, only ($E_{\mathrm{inh}}^{a}$ > *E*_a_) can be adsorbed on the electrode area of the metallic sample via electrostatic bonds (the physisorption mode). However, the activation energy of the inhibitory resin is lower than that of the 0.5 M H_2_SO_4_ alone ($E_{\mathrm{inh}}^{a}$ < 𝐸_a_), which could be adsorbed on the metallic sample area by the covalent bonds (the chemisorption mode). A careful analysis of the data illustrated in Table 5 reveals that the activation energy value of the TGP is higher than that of the 0.5 M H_2_SO_4_ alone. These data indicate that the tested inhibitory resin could be adsorbed via the physico-chemical adsorption mode on the surface of the steel electrode. In addition, Figure 6 illustrates the variations in the ln(*i*_corr_/*T*) as a function of the 1000/T in an aggressive environment for MS uninhibited and inhibited with 10^−3^ M of the investigated inhibitory resin. In addition, the slopes of the straight lines were investigated in order to obtain the values of the activation standard enthalpy; meanwhile, the intercept allows us to determine the activation standard entropy. Furthermore, according to the results illustrated in Table 5, the positive value of ∆*H*_a_ reflects the endothermic nature of the surface of the steel electrode and the dissolution of the corrosion inhibition process. In addition, the negative signs of ∆*S*_a_ in the aggressive environment for uninhibited MS mean that the activated TGP molecule was in a lower order state compared to the initial step. However, in the presence of 10^−3^ M of TGP inhibitory resin, the activation standard entropy (∆*S*_a_) is positive; this result indicates that the activated molecule was in a higher order state compared to the initial step. Furthermore, according to the data listed in Table 5, the activation standard entropy value with 10^−3^ M of TGP is higher than that of the uninhibited MS (0.5 M H_2_SO_4_ alone); thus, there is an increase in the disorder during the adsorption process.

### 3.5. DFT Results

**Figure 7:**
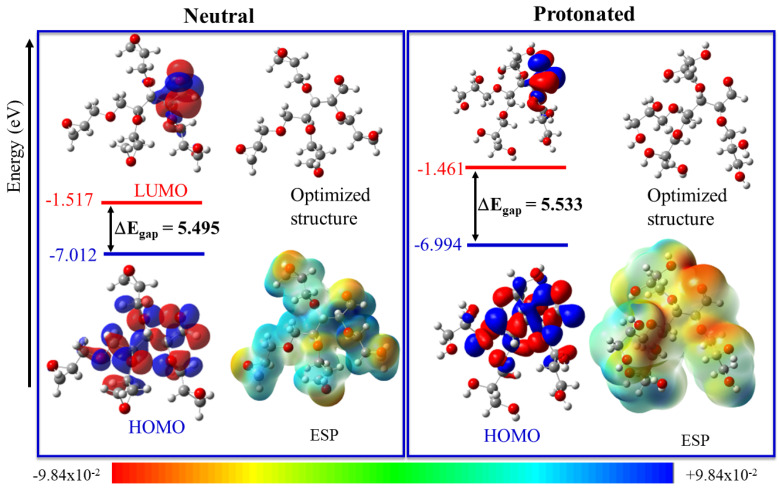
Optimized structures, 3D-plot electrostatic potential map and 3D-Iso-surface densities of HOMOs and LUMOs, and the energies and energy gaps (in eV) (Isovalue: 0.02 au) of the investigated epoxy resin in its neutral form (**left**) and protonated form (**right**) in an aqueous solution using a B3LYP/6-31+G(d,p) level of theory with a PCM solvation model.

In order to confirm the data obtained during the experimental part, the DFT descriptors, namely *E*_HOMO_, *E*_LUMO_, ∆*E*_gap_, *χ*, *μ*, *S*, *ω*, Δ*N* and ∆*E*_b−d_, were calculated so as to study the inhibitory efficacy of the employed TGP inhibitory resin used to protect the mild steel electrode area from corrosion in the 0.5 M H_2_SO_4_ solution. For the sake of comparison, the neutral and protonated forms of the investigated resin were used in an aqueous environment. Figure 7 illustrates the optimized structures, the HOMO and LUMO energy molecular orbitals, electrostatic potential maps (ESP) and the energy gap profiles of the neutral epoxy resin (left) and its protonated form (right). In addition, the data obtained are displayed in Table 6.

The distribution of the HOMO surface of the neutral/protonated forms on all of the parts of the molecules, except the oxirane rings, implies that the carbonyl oxygen atoms and the oxygen ether group atoms are able to donate their electrons to the vacant d-orbital of the metal. However, the LUMO surface is mostly distributed on the carbonyl function and its neighboring carbon atoms, implying that this part of the epoxide resin has the ability to accept electrons from the occupied d-orbital of the metallic substrates area. These data are also supported by the electrostatic potential map.

As is known, *E*_HOMO_ is the most commonly used quantum chemical descriptor, and is associated with the tendency to donate electrons to the vacant d-orbitals of the metallic area. The high values of the *E*_HOMO_ suggest the high propensity of electrons to transfer to the lowest virtual d-orbital of the acceptor [37]. On the other hand, *E*_LUMO_ indicates that inhibitors can accept electrons from the vacant d-orbital of the metallic area. It has been outlined that the lower that the *E*_LUMO_ of the molecule is, the greater its tendency to accept electrons from the donor. Therefore, the adsorptive mechanism can be supported by the donation and acceptance competence to/from the inhibitor/metal and can also help to protect the metallic area from corrosion. The chemical reactivity of the inhibitors also depends on the energy gap between the *E*_HOMO_ and *E*_LUMO_ [38]. The smaller the value of the energy gap, the more reactive the inhibitory resin is. Additionally, small hardness values and higher softness values also have a critical role in increasing the anticorrosive properties of the inhibitory resins. The energy gap of the investigated compounds is small enough compared to the other compounds, which makes it reactive and able to protect the metallic substrates from corrosion. It was also illustrated that when η > 0 or *ΔE*_b−d_ < 0, back-donation from the inhibitory resin to the metallic area is energetically favored [23]. The anticorrosive properties of the inhibitors are also affected by the higher values of the absolute electronegativity, which show that inhibitory resin cannot share electrons with others easily. The results in Table 6 show that the employed inhibitory resin has a comparable electronegativity value (4.264/4.228 eV (neutral/protonated), which enhances its inhibition efficiency. The positive values of the ΔN(0.108/0.113 (neutral/protonated)), which are lower than 3.6, suggest that the transfer of electron takes place between the inhibitory resin and the metallic surface [39]. The dipole moments of the synthesized resin in their neutral and protonated forms of 6.3 and 13.6 Debye, respectively, are higher than those of water of 1.8 Debye. This result displays the tendency of the inhibitory resin to expel water from the area of the metallic substrates and increase its inhibition efficiency. Furthermore, it was found that high values in the dipole moment shows a high value of polarizability, which is related to the inhibition efficacy of the inhibitory resin. As is known, if the resin easily polarizes, the tested resin can donate to or share electrons with the metallic substrates area.

Since we are dealing with the donation/acceptance of electrons to/from metal, it is helpful to estimate two important parameters that explain the metal–inhibitor interaction (Δ*E*_1_ and Δ*E*_2_) [38]. These descriptors are able to determine whether the electrons are transferred from the epoxide resin to the mild steel electrode area or vice versa. Δ*E*_1_ ($E_{LUMO}^{inh}-E_{HOMO\;}^{Fe}$) denotes the mechanism through which the electron is donated from the occupied d-orbitals of the Fe metallic samples area to the LUMO of the inhibitory resin. Meanwhile, ∆*E*_2_ ($E_{LUMO}^{Fe}-E_{HOMO}^{inh})$ denotes the mechanism through which the electrons are donated from the HOMO of the inhibitory resin to the unoccupied d-orbitals of the Fe metallic substrates area [18]. In this study, the *E*_HOMO_ and *E*_LUMO_ of iron were found, using the 88th Edition of the CRC Handbook of Chemistry and Physics, to be −7.9024 and −0.151 eV, respectively [40]. For the neutral/protonated form, the data displayed in Table 6 illustrates that the *ΔE*_1_ (6.368/6.441 eV) values are slightly lower than those of Δ*E*_2_ (6.861/6.843 eV). These results reveal that the electron’s transfer from the metal ion to the inhibitor is energetically favored.

**Figure 8:**
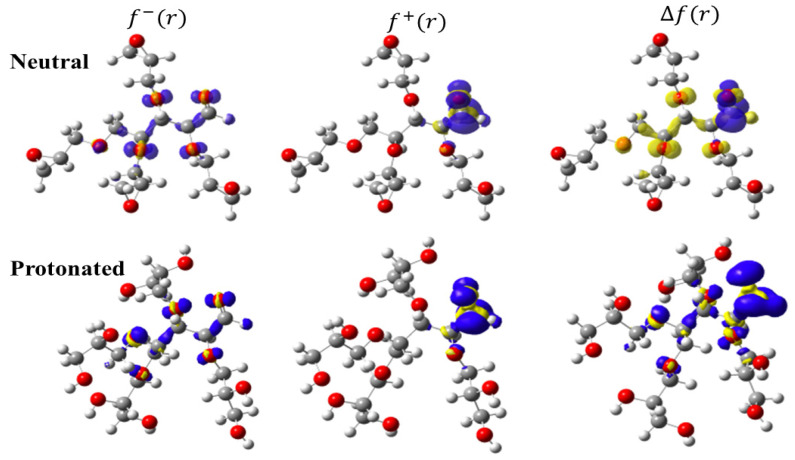
The 3D plots of the iso-surfaces of the electrophilic $(f^{-}\left(r\right)$ (**left**), nucleophilic $(f^{+}\left(r\right)$) (**middle**) and dual Fukui descriptor (**right**) of the TGP inhibitor, as found using the B3LYP/6-31+G(d,p)/H_2_O/PCM model of theory. Blue and negative lobes correspond to positive and negative values, respectively.

**Figure 9:**
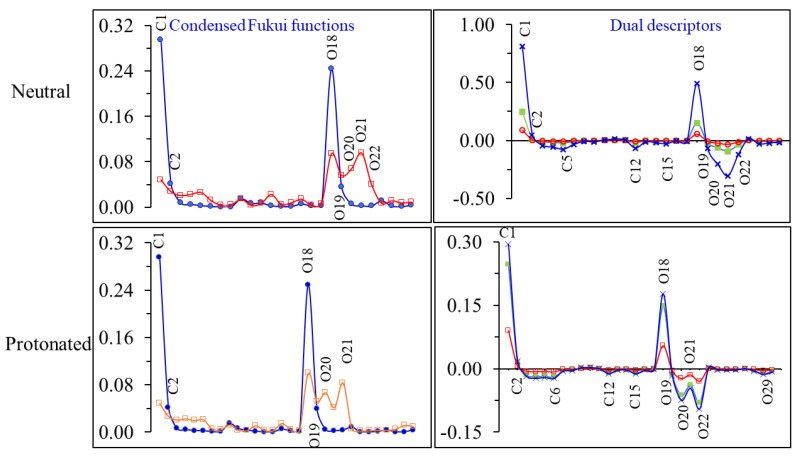
Condensed Fukui indices and the dual local descriptors (all values are in Hartree) of the TGP inhibitory resin. For the condensed Fukui functions: Open red and orange squares correspond to $f_{k}^{-},\;$ filled blue circles correspond to $f_{k}^{+}$. For dual descriptors: Filled green squares correspond to $f_{k}^{2}$, filled red squares correspond to Δ*s_k_* and blue x marks correspond to Δ$w_{k}$.

The 3D plots of the iso-area of the nucleophilic and electrophilic Fukui functions, and the dual Fukui descriptors of the employed inhibitory epoxide resin in its neutral and protonated forms in an aqueous environment with an iso value = 0.02 au and a density = 0.005 au, are displayed in Figure 8. A careful examination of the data illustrated in Figure 8 (in both forms) reveals that the electrophilic attack sites are more important compared to the nucleophilic attack sites, which might be explained by the presence of oxygen atoms. Figure 9 shows the condensed nucleophilic ($f_{k}^{+}$) and electrophilic ($f_{k}^{-}$) Fukui functions, the local softness ($S_{k}^{\pm }$), local electrophilicity ($\omega_{k}^{\pm }$) and their dual local descriptors ($f_{k}^{2}$, Δ*s_k_* and Δ$w_{k}$). All sets of numerical values are given in the supporting information (Tables S1 and S2) (note that the numbering system of the atoms is shown in Scheme 1). For both forms, the results reveal that the reactive nucleophilic attacks are produced by the carbonyl group (*C*_1_, *O*_18_, *C*_2_ and *C*_19_), whereas the more reactive sites for the electrophilic attacks are the oxygen heteroatoms (*O*_21_, *O*_18_ and *O*_21_ and *O*_20_) (Table 7).

### 3.6. Monte Carlo and Molecular Dynamic Approaches

Starting with the Fe(110) area makes it easy to compute the system’s adsorption energies. Applying Equation (9) to a molecule yields its adsorption energy, or *E*_ads_ [41]:

(9) $$E_{adsorption}=E_{\mathrm{Fe}{\left(110\right)}_{\left|\right|}inhibitor\;}-\left(\mathrm{Fe}\left(110\right)+E_{inhibitor}\right)\;$$

where $E_{\mathrm{Fe}{\left(110\right)}_{\left|\right|}inhibitor}$ is the total energy of the simulated system, *E_Fe_*, and *E_inhibitor_* are the total energy of the Fe(110) area and the corresponding free inhibitory resins, respectively.

**Figure 10:**
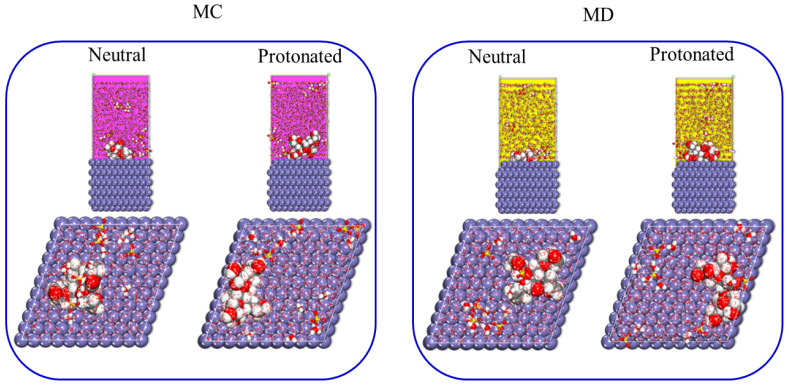
(**Left**): MC and (**Right**): MD poses the lowest adsorption configurations for inhibitory resin adsorption in the simulated aggressive environment on the Fe(110) area.

After the MC approaches were successful (Figure 10), a considerable amount of research was conducted on the adsorption geometry of the inhibitory resin to make sure that the results were valid. This was performed to make sure that the data were correct. The MC simulation’s ability to reach a state of equilibrium can be judged by comparing the energy values at the steady state to the energy values at the start of the simulation. The simulation had reached the point at which the system could operate with the least amount of energy needed to keep going. The actual arrangement of the adsorbent inhibitory resin is displayed in Figure 10. This picture is based on a simulated plane of the type Fe-110. During the MD technique, the inhibitor decorates the area of Fe(110) in a way that is almost parallel to how the inhibitory resin is oriented on the side that maximizes contact to its oxygen atoms. We think that the backbone of an inhibitory resin that is willing to stick to the surface atoms of the Fe(110) plane causes this adsorption pattern, as shown in Figure 10. This theory is supported by the evidence that has been gathered. The tendency of resins to bring their heteroatoms (O) and electron rings closer to the area makes it possible for those inhibitory resins to adsorb, and the fact that they can do so is what gives those epoxide resins their ability to adsorb [42].

**Figure 11:**
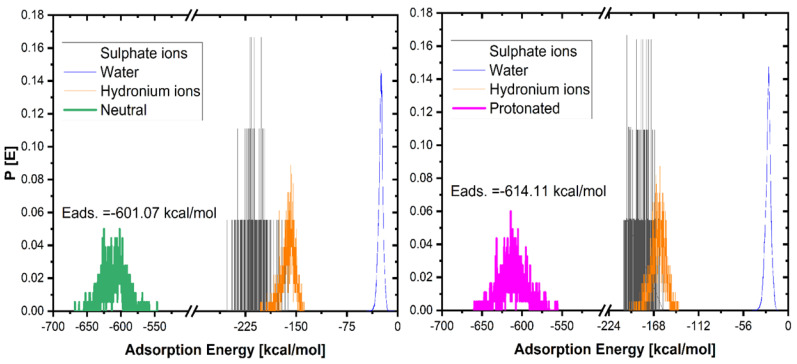
Distribution of the adsorption energy of the inhibitory resin employed in the simulated aggressive environment obtained using MC approaches.

**Figure 12:**
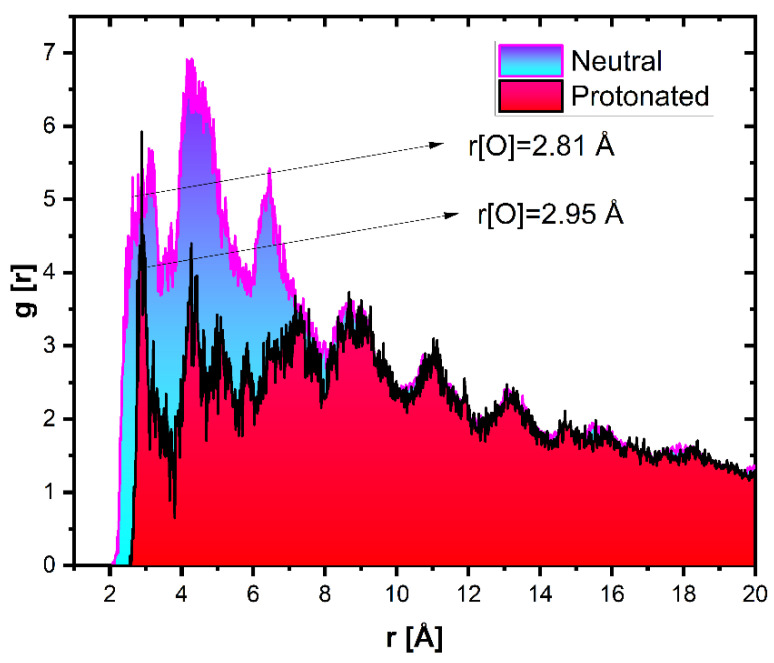
RDF oxygen atoms for the inhibitor (neutral and protonated) on the Fe(110) area accomplished using MD trajectory approach.

The adsorption of inhibitory resin onto the metallic substrates area offers data on the formation of the substantial Eads (Figure 11), which are visible on the metallic samples area. The inhibitory resin has an important adsorption interaction with the metallic area, owing to the extraordinarily high adsorption energies that it possesses. This activity, which is evidenced in the creation of a protective thin layer on the metallic substrates area, makes the electrode area of the metallic samples resistant to corrosion, which protects the metal’s original tate [26]. MD is widely considered to be a more specific illustration of the adsorption dynamics. In addition, after running the NVT simulation for several hundreds of picoseconds, it is abundantly obvious that the inhibitory resins depicted in Figure 11 adopt a configuration that is somewhat flat on the sides of the oxiran systems onto the metallic substrates area. Because of this, the inhibitory resins are powerfully absorbed onto the iron area. In addition, the radial distribution function (RDF) approach of the MD trajectory carried out during corrosion simulations is a simple technique that may be used to investigate the anticorrosion inhibitor that is adsorbed on the metallic substrates area (Figure 12). These results were obtained by running the simulations. This approach is uncomplicated and does not entail any tricky steps [43]. In MD simulations, the RDF (g) is a great way to assess the interaction between molecules and surfaces of iron [30]. The peak for chemisorption is between 1 and 3.5 Å, while the peak for physisorption is higher than 3.5 Å [28]. Figure 12 shows the radial distribution function of the inhibitor. This shows that the distance between the Fe (surface) and the inhibitor (2.81 and 2.95 Å for neutral and protonated inhibitor, respectively) is less than 3.5 Å. The negative energy value and the RDF peaks, indicates that the inhibitory resin has a significant effect on the metallic substrates area. A careful analysis of the data illustrated in Figure 12 reveals that the inhibitory resin mostly interacts with the iron area via oxygen atoms.

## 4. Conclusions

The corrosion inhibition of the employed inhibitory resin has been investigated via electrochemical analyses, morphology and computational approaches. PDP and EIS techniques illustrated that the inhibitory resin possessed high inhibitory efficacies at its optimum concentrations, such as at 88.6% (PDP) and 85.6% (EIS). SEM/EDS analyses revealed the potential ability of inhibitory resin to protect a mild steel surface in 0.5 M H_2_SO_4_. Data obtained using the PDP technique suggest that the employed epoxide resin can be adsorbed strongly in the metallic substrates area, as an anodic-type inhibitor. Additionally, the DFT calculation gives further data about the reactivity and energetic properties of the interaction between the inhibitory resin and the iron area. According to the data obtained using the MC and MD approaches, the inhibitory resin has a somewhat flat-adsorbed configuration on the side walls of the resin, which maximizes its contact with the oxygen heteroatoms. The concept that the inhibitory resin contains important negative adsorption energies is given further credence by the evidence that was gathered through experimental testing.

## Figures and Tables

**Table 1:** PDP parameters for MS in 0.5 M H_2_SO_4_, with and without inhibitory resin at different concentrations.

	Conc. (M)	−*E*_corr_ (mV/Ag/AgCl)	*i*_corr_ (µA cm^−2^)	−*β*_c_ (mV dec^−1^)	*β*_a_ (mV dec^−1^)	*ƞ*_PDP_ (%)
Uninhibited	0.5	451	1850	99	121	-
Inhibited with TGP	10^−6^	560	472	147	90	74.4
	10^−5^	563	469	146	86	74.6
	10^−4^	548	222	155	79	88.0
	10^−3^	556	211	148	71	88.6

**Table 2:** EIS parameters for MS immersed in 0.5 M H_2_SO_4_ medium, uninhibited and inhibited by different concentrations of TGP.

	Conc. (M)	*R*_e_ (Ω cm^2^)	*R*_ct_ (Ω cm^2^)	*R*_L_ (Ω cm^2^)	*L* (H)	*Q* (µF s^n−1^)	*n*_dl_	*θ*	*ƞ*_EIS_ (%)
Uninhibited	0.5	1.6	11.7	2.17	0.48	430	0.830	-	-
Inhibited with TGP	10^−6^	2.2	39.0	6.45	0.60	195	0.811	0.70	70.0
	10^−5^	2.1	40.97	7.53	0.71	175	0.828	0.714	71.4
	10^−4^	2.2	78.27	17.79	2.55	148	0.762	0.850	85.0
	10^−3^	2.1	81.37	22.35	3.47	140	0.794	0.856	85.6

**Table 3:** EDS analysis of MS surface after immersion in 0.5 M H_2_SO_4_ at each point with and without 10^−3^ M of TGP.

	Each Point	C (%)	O (%)	S (%)	Cl (%)	Fe (%)
0.5 M H_2_SO_4_	Spc_001	3.47	22.92	3.43	0.24	69.93
	Spc_002	4.47	34.05	7.71	0.21	53.56
	Spc_003	3.66	22.62	5.89	0.24	67.60
10^−3^ M of TGP	Spc_001	5.11	24.63	5.82	0.30	64.14
	Spc_002	5.59	17.66	2.28	0.39	74.07
	Spc_003	5.04	9.51	0.97	0.22	84.26

**Table 4:** Adsorption parameters of the TGP for MS in 0.5 M H_2_SO_4_ at 298 K.

	K_ads_ (L/mol)	*R*²	Δ*G*_ads_ (KJ/mol)
TGP	1,265,760.29	1	−44.74

**Table 5:** Electrochemical and kinetic parameters of MS uninhibited and inhibited in 0.5M H_2_SO_4_ with inhibitory TGP resin (10^−3^ M) at different temperatures.

*T* K	−*E*_corr_ mV_Ag/AgCl_	*i*_corr_ µA cm^−2^	−*β*_c_ mV dec^−1^	$\eta_{\mathrm{PC}}\%$	*E*_a_ kJ mol^−1^	Δ*H*_a_ kJ mol^−1^	−Δ*S*_a_ J mol^−1^ K^−1^
MS substrates/0.5 M H_2_SO_4_							
298	451	1850	99	-	15	12.5	140.5
308	453	2250	92	-			
318	449	2480	96	-			
328	442	3340	102	-			
TGP/MS substrates/0.5 M H_2_SO_4_							
298	556	211	148	88.6	28.32	25.8	113.6
308	540	325	137	85.5			
318	549	435	132	82.4			
328	545	614	152	81.6			

**Table 6:** Various DFT parameters for the epoxy resin (neutral and protonated forms).

	Neutral	Protonated
Total energy, E(N) (Hartree)	−1340.3180	−1646.2206
Dipole moment, μ, (Dybe)	6.4	13.6
*E*_HOMO_ (eV)	−7.012	−6.994
*E*_LUMO_ (eV)	−1.517	−1.461
Δ*E*_gap_ (eV)	5.495	5.533
*I_v_* (eV)	6.949	6.934
*A_v_* (eV)	1.517	1.465
*χ* (eV)	4.233	4.200
*η* (eV)	2.716	2.735
*S* (eV^−1^)	0.368	0.366
*ω* (eV)	3.299	3.225
Δ*N*	0.108	0.113
Δ*E_b−d_* (eV)	−0.679	−0.684
Δ*E_1_* (eV)	6.386	6.441
Δ*E_2_* (eV)	6.861	6.843

**Table 7:** Fukui functions and the dual local descriptors.

Atoms	$f_{k}^{+}$	$f_{k}^{-}$	$f_{k}^{2}$	$s_{k}^{+}$	$s_{k}^{-}$	Δs	$w_{k}^{+}$	$w_{k}^{+}$	Δw
C1	0.042	0.027	0.015	0.411	0.263	0.149	3.674	2.347	1.327
C10	0.296	0.049	0.247	2.908	0.479	2.429	25.983	4.281	21.703
O11	0.249	0.100	0.149	2.444	0.979	1.466	21.843	8.746	13.097
O13	0.039	0.052	−0.013	0.383	0.512	−0.130	3.419	4.580	−1.160
O14	0.004	0.083	−0.080	0.034	0.816	−0.782	0.308	7.296	−6.988
O21	0.004	0.067	−0.063	0.041	0.656	−0.615	0.369	5.863	−5.494
O25	0.002	0.041	−0.039	0.024	0.405	−0.382	0.211	3.621	−3.411

## Data Availability

The data that support the findings of this study are available from the corresponding author upon reasonable request.

## Supplementary Materials

The following supporting information can be downloaded at: https://www.mdpi.com/article/10.3390/polym15081967/s1, Figure S1: Langmuir adsorption isotherm model of TGP for MS in 0.5 M H _2_SO_4_ at 298 K; Table S1: Hirshfeld charges, condensed Fukui functions and condensed dual descriptors for the neutral form of the TGP inhibitor molecules as obtained at the B3LYP/6-31G(d,p)/H_2_O/PCM model of theory. Units used below are “e” (elementary charge); Table S2: Hirshfeld charges, condensed Fukui functions and condensed dual descriptors for the protonated form of the TGP inhibitor molecules as obtained at the B3LYP/6-31G(d,p)/H_2_O/PCM model of theory. Units used below are “e” (elementary charge).
