# Supplementary material for: A Combined Experimental and Computational (DFT, RDF, MC and MD) Investigation of Epoxy Resin as a Potential Corrosion Inhibitor for Mild Steel in a 0.5 M H2SO4 Environment
Source: Polymers (Basel). 2023 Apr 21;15(8):1967. doi: 10.3390/polym15081967 (PMC10145058; doi:10.3390/polym15081967)
Supplement: Supplementary file 1 [file polymers-15-01967-s001.zip › polymers-2309030-supplementary.pdf]

A combined experimental and computational (DFT, RDF, MC and MD) investigation of epoxy resin as a potential corrosion inhibition for mild steel in 0.5 M H_2SO4 environment

Rachid Hsissou[1,2*], Khadija Dahmani[2], Anouar El Magri[3], Abdelfettah Hmada[4], Zaki Safi[5], Nadia Dkhireche[4], Mouhsine Galai[4], Nuha Wazzan[6], Avni Berisha[7]

[1] Laboratory of Organic Chemistry, Bioorganic and Environment, Chemistry Department, Faculty of Sciences, Chouaib Doukkali University, El Jadida, Morocco.

[2] Laboratory of Organic Chemistry, Catalysis and Environment, Department of Chemistry, Faculty of Sciences, Ibn Tofail University, BP 242, 14000, Kenitra, Morocco.

[3] Euromed Polytechnic School, Euromed Research Center, Euromed University of Fez, 30 000, Fez, Morocco.

[4] Laboratory of Advanced Materials and Process Engineering, Faculty of Sciences, Ibn Tofail University, BP 242, 14000 Kenitra, Morocco.

[5] Al Azhar University-Gaza, Chemistry Department, Faculty of Science, P.O Box 1277, Gaza, Palestine.

[6] King Abdulaziz University, Chemistry Department, Faculty of Science, P.O Box 42805, Jeddah, 21589, Saudi Arabia.

[7] Department of Chemistry, Faculty of Natural and Mathematics Science, University of Prishtina, 10000 Prishtina, Kosovo

Corresponding author: Rachid Hsissou Email: r.hsissou@gmail.com

Supporting information

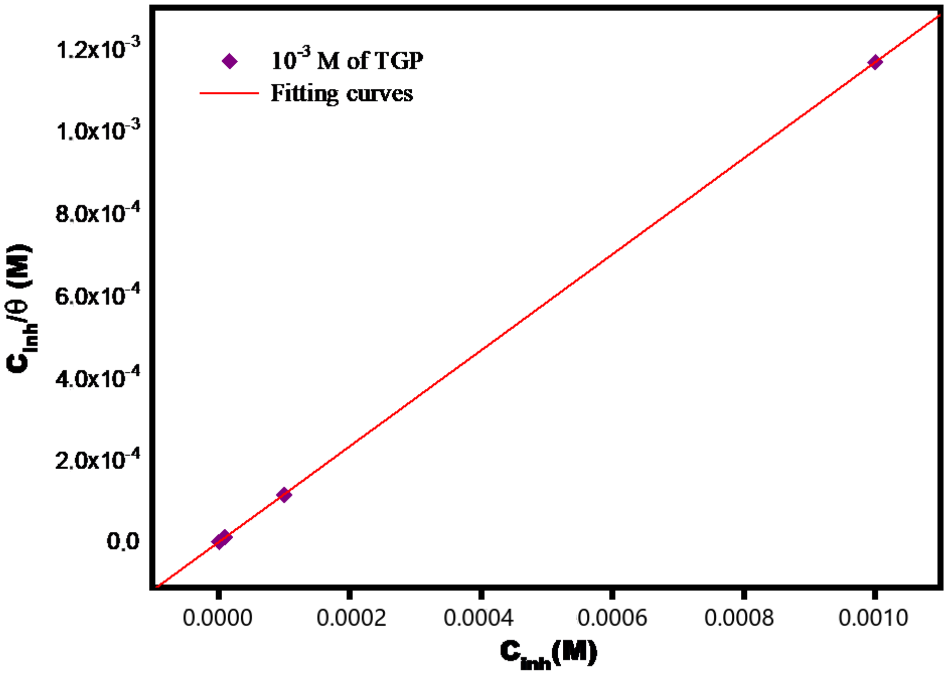

Figure. S1. Langmuir adsorption isotherm model of TGP for MS in 0.5 M H_2SO_4 at 298 K

Table S1. Hirshfeld charges, condensed Fukui functions and condensed dual descriptors for the neutral form of the TGP inhibitor molecules as obtained at the B3LYP/6-31G(d,p)/H_2O/PCM model of theory. Units used below are "e" (elementary charge)

atoms	q_N	q_{N+1}	q_{N-1}	f_k^+	f_k^-	f_k^2	s_k^+	s_k^-	Δs_k	w_k^+	w_k^+	Δw_k
C1	0.1513	-0.1433	0.1993	0.2945	0.0480	0.2465	0.1084	0.0177	0.0907	0.9716	0.1584	0.8132
C2	0.0502	0.0090	0.0776	0.0412	0.0274	0.0138	0.0152	0.0101	0.0051	0.1359	0.0904	0.0455
C3	0.0552	0.0478	0.0757	0.0074	0.0205	-0.0131	0.0027	0.0075	-0.0048	0.0244	0.0676	-0.0432
C4	0.0511	0.0463	0.0738	0.0049	0.0227	-0.0178	0.0018	0.0084	-0.0066	0.0162	0.0749	-0.0587
C5	0.0180	0.0151	0.0439	0.0028	0.0259	-0.0231	0.0010	0.0095	-0.0085	0.0092	0.0854	-0.0762
C6	0.0283	0.0270	0.0401	0.0012	0.0118	-0.0106	0.0004	0.0043	-0.0039	0.0040	0.0389	-0.0350
C7	0.0363	0.0360	0.0393	0.0003	0.0030	-0.0027	0.0001	0.0011	-0.0010	0.0010	0.0099	-0.0089
C8	0.0123	0.0119	0.0164	0.0004	0.0041	-0.0037	0.0001	0.0015	-0.0014	0.0013	0.0135	-0.0122
C9	0.0331	0.0184	0.0471	0.0147	0.0140	0.0007	0.0054	0.0052	0.0003	0.0485	0.0462	0.0023
C10	0.0374	0.0309	0.0404	0.0065	0.0030	0.0035	0.0024	0.0011	0.0013	0.0214	0.0099	0.0115
C11	0.0174	0.0100	0.0239	0.0074	0.0065	0.0009	0.0027	0.0024	0.0003	0.0244	0.0214	0.0030
C12	0.0216	0.0192	0.0439	0.0024	0.0223	-0.0199	0.0009	0.0082	-0.0073	0.0079	0.0736	-0.0657
C13	0.0339	0.0327	0.0387	0.0012	0.0049	-0.0037	0.0004	0.0018	-0.0014	0.0040	0.0162	-0.0122
C14	0.0104	0.0093	0.0179	0.0012	0.0075	-0.0063	0.0004	0.0028	-0.0023	0.0040	0.0247	-0.0208
C15	0.0279	0.0223	0.0420	0.0056	0.0141	-0.0085	0.0021	0.0052	-0.0031	0.0185	0.0465	-0.0280
C16	0.0356	0.0334	0.0391	0.0022	0.0035	-0.0013	0.0008	0.0013	-0.0005	0.0073	0.0115	-0.0043
C17	0.0121	0.0093	0.0178	0.0028	0.0057	-0.0029	0.0010	0.0021	-0.0011	0.0092	0.0188	-0.0096
O18	-0.2482	-0.4921	-0.1539	0.2439	0.0942	0.1497	0.0898	0.0347	0.0551	0.8046	0.3108	0.4939
O19	-0.1591	-0.1951	-0.1033	0.0360	0.0558	-0.0198	0.0132	0.0205	-0.0073	0.1188	0.1841	-0.0653
O20	-0.1594	-0.1649	-0.0916	0.0055	0.0678	-0.0623	0.0020	0.0250	-0.0229	0.0181	0.2237	-0.2055
O21	-0.1726	-0.1753	-0.0772	0.0026	0.0954	-0.0928	0.0010	0.0351	-0.0342	0.0086	0.3147	-0.3061
O22	-0.1647	-0.1666	-0.1250	0.0019	0.0396	-0.0377	0.0007	0.0146	-0.0139	0.0063	0.1306	-0.1244
O23	-0.2252	-0.2365	-0.2183	0.0113	0.0069	0.0044	0.0042	0.0025	0.0016	0.0373	0.0228	0.0145
O24	-0.2256	-0.2275	-0.2144	0.0018	0.0113	-0.0095	0.0007	0.0042	-0.0035	0.0059	0.0373	-0.0313
O25	-0.2243	-0.2251	-0.2168	0.0008	0.0075	-0.0067	0.0003	0.0028	-0.0025	0.0026	0.0247	-0.0221
O26	-0.2229	-0.2269	-0.2138	0.0039	0.0091	-0.0052	0.0014	0.0033	-0.0019	0.0129	0.0300	-0.0172

Table S2. Hirshfeld charges, condensed Fukui functions and condensed dual descriptors for the protonated form of the TGP inhibitor molecules as obtained at the B3LYP/6-31G(d,p)/H_2O/PCM model of theory. Units used below are "e" (elementary charge)

atoms	q_N	q_{N+1}	q_{N-1}	f_k^+	f_k^-	f_k^2	s_k^+	s_k^-	Δs_k	w_k^+	w_k^+	Δw_k
C1	0.1478	-0.1478	0.1965	0.2956	0.0487	0.2469	0.1082	0.0178	0.0904	0.3522	0.0580	0.2941
C2	0.0511	0.0093	0.0778	0.0418	0.0267	0.0151	0.0153	0.0098	0.0055	0.0498	0.0318	0.0180
C3	0.0509	0.0450	0.0714	0.0058	0.0206	-0.0148	0.0021	0.0075	-0.0054	0.0069	0.0245	-0.0176
C4	0.0470	0.0429	0.0696	0.0041	0.0226	-0.0185	0.0015	0.0083	-0.0068	0.0049	0.0269	-0.0220
C5	0.0169	0.0144	0.0365	0.0024	0.0196	-0.0172	0.0009	0.0072	-0.0063	0.0029	0.0234	-0.0205
C6	0.0148	0.0126	0.0361	0.0022	0.0212	-0.0190	0.0008	0.0078	-0.0070	0.0026	0.0253	-0.0226
C7	0.0500	0.0492	0.0555	0.0008	0.0055	-0.0047	0.0003	0.0020	-0.0017	0.0010	0.0066	-0.0056
C8	0.0248	0.0242	0.0289	0.0006	0.0041	-0.0035	0.0002	0.0015	-0.0013	0.0007	0.0049	-0.0042
C9	0.0309	0.0166	0.0429	0.0143	0.0120	0.0023	0.0052	0.0044	0.0008	0.0170	0.0143	0.0027
C10	0.0473	0.0414	0.0508	0.0059	0.0035	0.0024	0.0022	0.0013	0.0009	0.0070	0.0042	0.0029
C11	0.0283	0.0249	0.0310	0.0033	0.0027	0.0006	0.0012	0.0010	0.0002	0.0039	0.0032	0.0007
C12	0.0193	0.0181	0.0302	0.0012	0.0109	-0.0097	0.0004	0.0040	-0.0036	0.0014	0.0130	-0.0116
C13	0.0504	0.0499	0.0533	0.0004	0.0029	-0.0025	0.0001	0.0011	-0.0009	0.0005	0.0035	-0.0030
C14	0.0234	0.0231	0.0260	0.0003	0.0026	-0.0023	0.0001	0.0010	-0.0008	0.0004	0.0031	-0.0027
C15	0.0189	0.0141	0.0335	0.0048	0.0146	-0.0098	0.0018	0.0053	-0.0036	0.0057	0.0174	-0.0117
C16	0.0522	0.0503	0.0567	0.0019	0.0045	-0.0026	0.0007	0.0016	-0.0010	0.0023	0.0054	-0.0031
C17	0.0185	0.0175	0.0209	0.0010	0.0024	-0.0014	0.0004	0.0009	-0.0005	0.0012	0.0029	-0.0017
O18	-0.2585	-0.5071	-0.1590	0.2485	0.0995	0.1490	0.0910	0.0364	0.0545	0.2960	0.1185	0.1775
O19	-0.1572	-0.1961	-0.1051	0.0389	0.0521	-0.0132	0.0142	0.0191	-0.0048	0.0463	0.0621	-0.0157
O20	-0.1599	-0.1641	-0.0932	0.0042	0.0667	-0.0625	0.0015	0.0244	-0.0229	0.0050	0.0795	-0.0745
O21	-0.1694	-0.1718	-0.1282	0.0024	0.0412	-0.0388	0.0009	0.0151	-0.0142	0.0029	0.0491	-0.0462
O22	-0.1678	-0.1713	-0.0849	0.0035	0.0830	-0.0795	0.0013	0.0304	-0.0291	0.0042	0.0989	-0.0947
O23	-0.2596	-0.2677	-0.2539	0.0080	0.0057	0.0023	0.0029	0.0021	0.0008	0.0095	0.0068	0.0027
O24	-0.2440	-0.2441	-0.2418	0.0001	0.0021	-0.0020	0.0000	0.0008	-0.0007	0.0001	0.0025	-0.0024
O25	-0.2491	-0.2493	-0.2464	0.0003	0.0027	-0.0024	0.0001	0.0010	-0.0009	0.0004	0.0032	-0.0029
O26	-0.2421	-0.2431	-0.2390	0.0010	0.0032	-0.0022	0.0004	0.0012	-0.0008	0.0012	0.0038	-0.0026
O27	-0.2315	-0.2347	-0.2285	0.0031	0.0030	0.0001	0.0011	0.0011	0.0000	0.0037	0.0036	0.0001
O28	-0.2444	-0.2448	-0.2397	0.0004	0.0047	-0.0043	0.0001	0.0017	-0.0016	0.0005	0.0056	-0.0051
O29	-0.2559	-0.2564	-0.2445	0.0004	0.0114	-0.0110	0.0001	0.0042	-0.0040	0.0005	0.0136	-0.0131
O30	-0.2490	-0.2525	-0.2391	0.0035	0.0099	-0.0064	0.0013	0.0036	-0.0023	0.0042	0.0118	-0.0076
